# Kernel-PCA data integration with enhanced interpretability

**DOI:** 10.1186/1752-0509-8-S2-S6

**Published:** 2014-03-13

**Authors:** Ferran Reverter, Esteban Vegas, Josep M Oller

**Affiliations:** 1 Department of Statistics, University of Barcelona, Diagonal, 643, 08028 Barcelona, Spain

**Keywords:** Dimensionality reduction, Kernel PCA, Data integration, Interpretability

## Abstract

**Background:**

Nowadays, combining the different sources of information to improve the biological knowledge available is a challenge in bioinformatics. One of the most powerful methods for integrating heterogeneous data types are kernel-based methods. Kernel-based data integration approaches consist of two basic steps: firstly the right kernel is chosen for each data set; secondly the kernels from the different data sources are combined to give a complete representation of the available data for a given statistical task.

**Results:**

We analyze the integration of data from several sources of information using kernel PCA, from the point of view of reducing dimensionality. Moreover, we improve the interpretability of kernel PCA by adding to the plot the representation of the input variables that belong to any dataset. In particular, for each input variable or linear combination of input variables, we can represent the direction of maximum growth locally, which allows us to identify those samples with higher/lower values of the variables analyzed.

**Conclusions:**

The integration of different datasets and the simultaneous representation of samples and variables together give us a better understanding of biological knowledge.

## Background

With the recent rapid advancements in high-throughput technologies, such as next generation sequencing, array comparative hybridization and mass spectrometry, databases are increasing in both the amount and the complexity of the data they contain. One of the main goals of mining this type of data is to visualize the relationships between biological variables that are involved [1]. For instance, visualizing gene expression guides the process of finding genes with similar expression patterns. However, due to the number of genes involved, it is more effective to display the data by means of a low-dimensional plot. Here we focus on the problem of reducing dimensionality and the interpretability of the resulting data representations.

Principal component analysis (PCA) has a very long history and is known to be a very powerful tool in the linear case. PCA is used as a visualization tool for the analysis of microarray data [2] and [3]. However, the sample space that many research problems deal with is considered nonlinear in nature; for example, the sample space of microarray data. One reason for this nonlinearity might be that the interactions of the genes are not completely understood. Many biological pathways are still not fully understood. So, it is quite naive to assume that genes are connected in a linear fashion. Following this line of thought, research into reducing the nonlinear dimensionality for microarray gene expression data has increased. Finding methods that can handle such data is of great importance if we are to glean as much information as possible from them.

Kernel representation offers an alternative to nonlinear functions by projecting the data into a high-dimensional feature space, which increases the computational power of linear learning machines [4] and [5]. Kernel methods enable us to construct different nonlinear versions of any algorithm which can be expressed solely in terms of dot products; this is known as the kernel trick. Kernel machines can be used to implement several learning algorithms but the interpretability of the resultant output representations may be cumbersome, because input variables are only handled implicitly [6].

Nowadays, combining multiple sources of data to improve the biological knowledge available is a challenging task in bioinformatics. Data analysis of different sources of information is not simply a matter of adding the analysis of each separate dataset; instead it consists of the simultaneous analysis of multiple variables in the different datasets [7].

Some of the most powerful methods for integrating heterogeneous data types are kernel-based methods [8] and [9]. We can describe kernel-based data integration approaches as using two basic steps. Firstly, the right kernel is chosen for each data set. Secondly, the kernels from the different data sources are combined to give a complete representation of the available data for a given statistical task. Basic mathematical operations such as multiplication, addition, and exponentiation preserve properties of kernel matrices and hence produce valid kernels. The simplest approach is to use positive linear combinations of the different kernels.

In this work, we analyze the integration of data from several sources of information using kernel PCA, from the point of view of reducing dimensionality and extending previous results [10]. Moreover, we improve kernel PCA interpretability by adding to the plot the representation of the input variables that belong to any dataset. In particular, for each input variable or linear combination of input variables, we can represent the direction of maximum growth locally, which allows us to identify those samples with higher/lower values of the variables analyzed. Therefore the integration of different datasets and the simultaneous representation of samples and variables together give us a better understanding of biological knowledge. This paper starts by briefly reviewing the notion of kernel PCA (Section 2). Section 3 contains our main results: a set of procedures to enhance the interpretability of kernel PCA when multiple datasets are analyzed simultaneously. We then present our results and apply them in parallel to analyze a nutrigenomic study in mouse [11].

## Results and discussion

Kernel methods enable us to construct different nonlinear versions of any algorithm which can be expressed solely in terms of dot products, this is the case of kernel PCA. Kernel PCA can be used to reduce dimensionality, thereby improving on linear PCA, but the interpretability of the output representations may be cumbersome because the input variables are only handled implicitly.

In this section, we propose a set of procedures to improve the interpretability of kernel PCA. The procedures are related to the following aspects:

• Representation of input variables.

• Data integration and representation of input variables.

• Representation of linear combinations of input variables.

• Revealing the interpretability of input variables.

To illustrate these procedures we use an example from metabolomics and genomics. The datasets come from a nutrigenomic study in mouse [11]. Forty mice were studied and two sets of variables were acquired: expressions of 120 genes measured in liver cells; and concentrations (in percentages) of 21 hepatic fatty acids (FAs) measured by gas chromatography. Biological units (mice) are cross-classified according to two factors: genotype, which can be wild-type (WT) or PPARα-deficient mice (PPAR); and diet, with 5 classes of diet in accordance with the FA composition.

The oils used for the experimental diet preparation were: corn and rapeseed oils (50:50), as the reference diet (ref); hydrogenated coconut oil, as a saturated FA diet (coc); sunflower oil, as an *ω*6 FA-rich diet (sun); linseed oil, as an *ω*3 FA-rich diet (lin); and corn, rapeseed and fish oils (42.5:42.5:15), as the fish diet. In the study, it cannot be assumed that variations in one set of variables cause variations in the other; we do not know a priori if changes in gene expression imply changes in FA concentrations or vice versa. Indeed, the nuclear receptor PPARα, which acts as a ligand-induced transcriptional regulator, is known to be activated by various FAs and to regulate the expression of several genes involved in FA metabolism. It should be noted that the main observations discussed in [11], which were extracted separately from the two datasets by both classical multidimensional tools (hierarchical clustering and PCA) and standard test procedures, are also highlighted by kernel PCA graphical representations.

### Representation of input variables

In order to achieve interpretability we add supplementary information into kernel PCA representations. We have developed a procedure to represent any given input variable on the subspace spanned by the eigenvectors of $\tilde{C}$ (see Methods).

We can consider that our observations are realizations of the random vector *X *= (*X*_1_*, ..., X_n_*). Then, to represent the prominence of the input variable *X_k _*in kernel PCA, we take a set of points of the form: **y **= **a **+ *s***e***_k _*∈ ℝ*^n^*, where **e***_k _*= (0, ..., 1, ..., 0) ∈ ℝ*^n^*, *s *∈ ℝ, and the *k*-th component is equal to 1 and the others are 0. Then, we can compute the projections of the image of these points, $\tilde{\phi }\left(y\right)$, onto the subspace spanned by the eigenvectors of $\tilde{C}$. Taking into account equation (8), the induced curve expressed in matrix form is given by the row vector:

$$\sigma {\left(s\right)}_{1\;\times \;r}^{k}=\left(Z_{s}^{T}-\frac{1}{m}1_{m}^{T}K\right)\left(I_{m}-\frac{1}{m}1_{m}1_{m}^{T}\right)\tilde{V},$$

where **Z***_s _*is in the form of (7).

In addition, we can represent directions of maximum growth of *σ^k^*(*s*) with respect the variable *X_k _*by projecting the tangent vector at *s *= 0. In matrix form, we have:

(1) $${\left(\frac{d\sigma^{k}}{ds}\right|}_{s=0}={\left(\frac{dZ_{s}^{T}}{ds}\right|}_{s=0}\left(I_{m}-\frac{1}{m}1_{m}1_{m}^{T}\right)\tilde{V},$$

With:

$${\left(\frac{dZ_{s}^{T}}{ds}\right|}_{s=0}={\left({\left(\frac{dZ_{s}^{1}}{ds}\right|}_{s=0},\ldots ,{\left(\frac{dZ_{s}^{m}}{ds}\right|}_{s=0}\right)}^{T},$$

and, using the chain rule:

(2) $${\left(\frac{dZ_{s}^{i}}{ds}\right|}_{s=0}\;=\;{\left(\frac{\partial K\left(y,x_{i}\right)}{\partial yk}\right|}_{y=a}.$$

In particular, let us consider the Gaussian radial basis function kernel: *k*(**x**, **z**) = exp(*-c *||**x **- **z**||^2^), with *c *> 0 a free parameter. Using the notation above, we have:

$$K\left(y,x_{i}\right)=\exp\left(-c{∥y-x_{i}∥}^{2}\right)=\exp\left(-c\overset{n}{\underset{t=1}{\sum }}{\left(y_{t}-x_{it}\right)}^{2}\right).$$

For the set of points of the form **y **= **a **+ *s***e***_k _*∈ ℝ*^n^*:

$${\left(\frac{dZ_{s}^{i}}{ds}\right|}_{s=0}\;=\;{\left(\frac{\partial K\left(y,x_{i}\right)}{\partial y_{k}}\right|}_{y=a}\;\;=-2cK\left(a,x_{i}\right)\left(a_{k}-x_{ik}\right).$$

In addition, if **a **= **x***_β _*(a training point) then:

$${\left(\frac{dZ_{s}^{i}}{ds}\right|}_{s=0}=-2cK\left(x_{\beta },x_{i}\right)\left(x_{\beta k}-x_{ik}\right).$$

**Figure 1:**
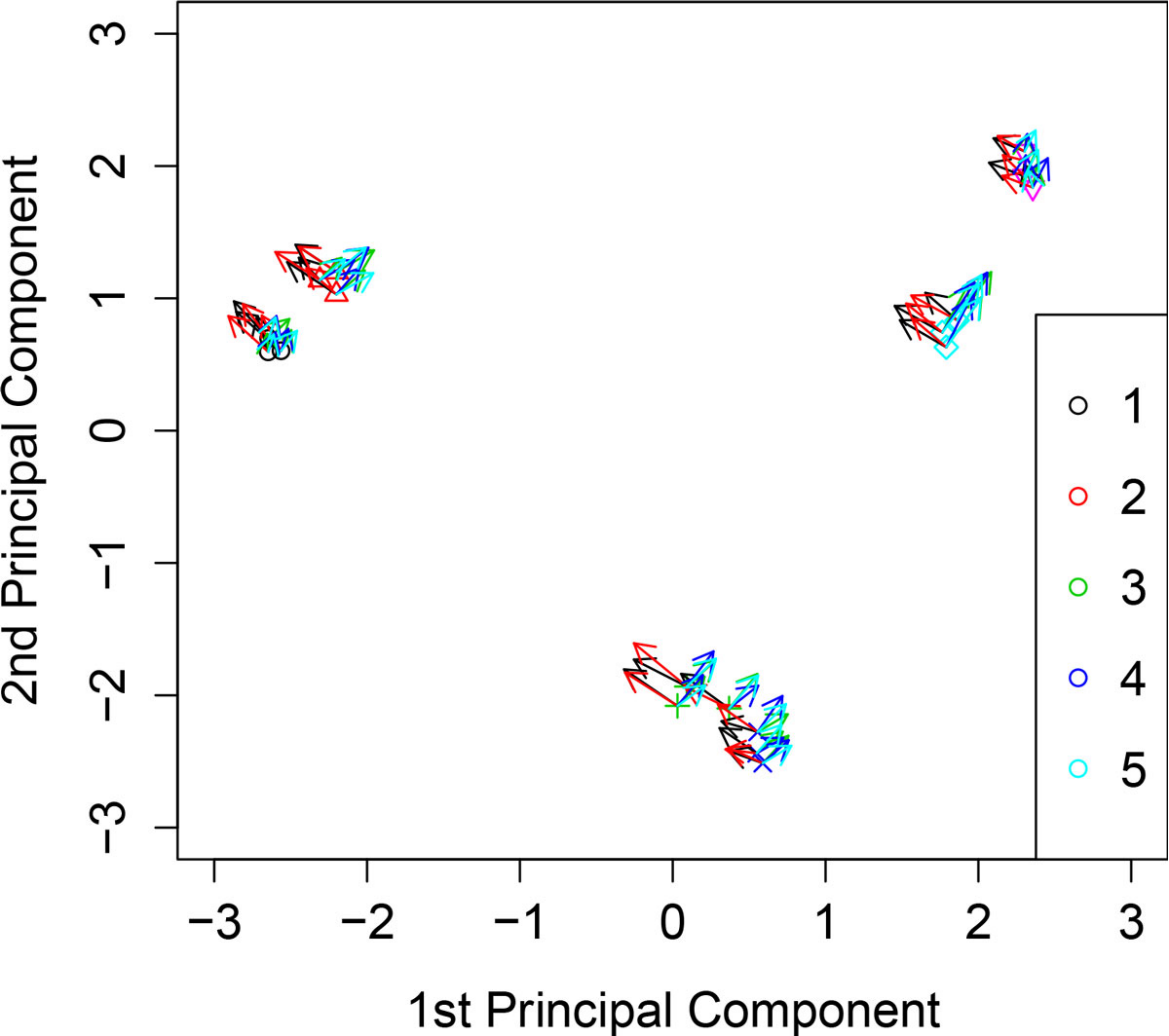
**Kernel PCA analyzing the toy example**. Variables are represented by vectors that indicate the direction of maximum growth in each variable.

To illustrate our procedure we introduce a toy example. We have generated a dataset which has 18 points in 6-dimensional space. Coordinates of the points are selected in order to distinguish 3 groups clearly separated. The group 1 has 6 points such that the sum of *X*_1 _and *X*_2 _coordinates is equal to 15 for each point. Moreover, in this group, there are 3 points such that the sum of *X*_3_, *X*_4 _and *X*_5 _is 0, and is equal to 6 for each the another 3 points. The group 2 has 6 points such that the sum of *X*_3_, *X*_4 _and *X*_5 _coordinates is equal to 0 for each point. Besides, in this group, there are 3 points such that the sum of *X*_1 _and *X*_2 _is 0, and is equal to -4 for each the another 3 points. Finally, the group 3 has 6 points such that the sum of *X*_1 _and *X*_2 _coordinates is equal to 0 for each point. Moreover, in this group, there are 3 points such that the sum of *X*_3_, *X*_4 _and *X*_5 _is 15, and is equal to 24 for each the another 3 points. All coordinates were perturbed with weak gaussian noise in order to introduce a small amount of variability inside each group. At each group the variable *X*_6 _is assigned randomly according to a Gaussian of mean zero and standard deviation 0.5. The configuration of the points is such that we expect that in reduction of dimension only the first dimensions are necessary to reveal the arrangement of the three groups. It can be seen in Figure 1 where the two leading components of kernel PCA are represented. We can see the group 1 (represented by triangles up and circles) on the negative part of the first principal axe, group 2 (represented by plus signs and by cross) in the central part and the group 3 (represented by diamonds and triangles down) on the positive part.

Figure 1 shows samples and the variables from *X*_1 _to *X*_5 _at each sample. Variables are represented by vectors that indicate the direction of maximum growth in each variable. In fact, we can see that the vectors point to those groups characterized by higher values in each variable. For instance, the variables *X*_1 _and *X*_2 _point to the group 1, and the variables *X*_3_, *X*_4_, and *X*_5 _point to the group 3.

**Figure 2:**
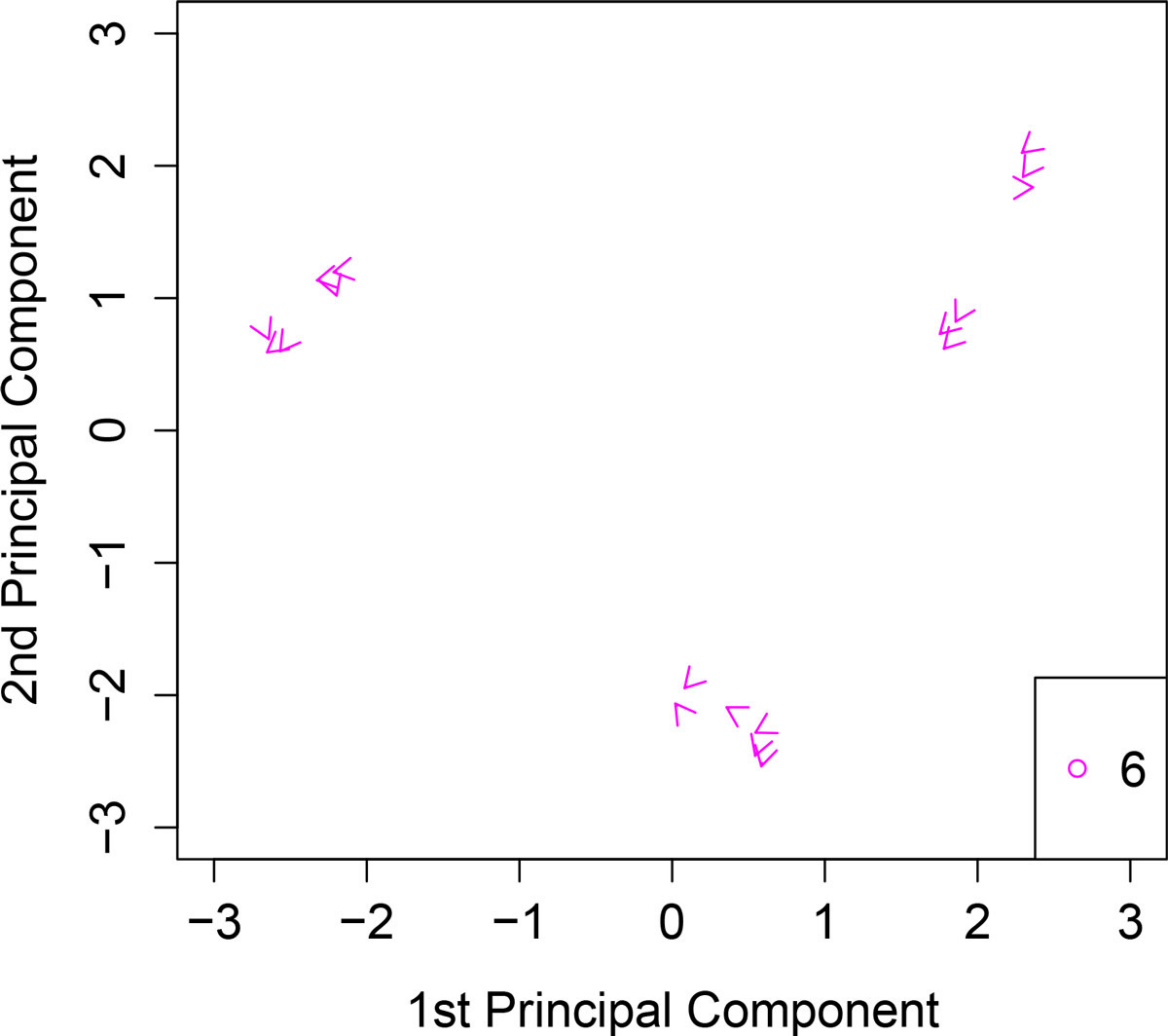
**Kernel PCA analyzing the toy example**. Variable *X*_6 _is poorly represented and the direction of maximum growth of this variable shows no trend to any group.

Figure 2 shows the variable *X*_6 _at each sample, we can observe that this variable is poorly represented and has no preferred direction towards any group.

**Figure 3:**
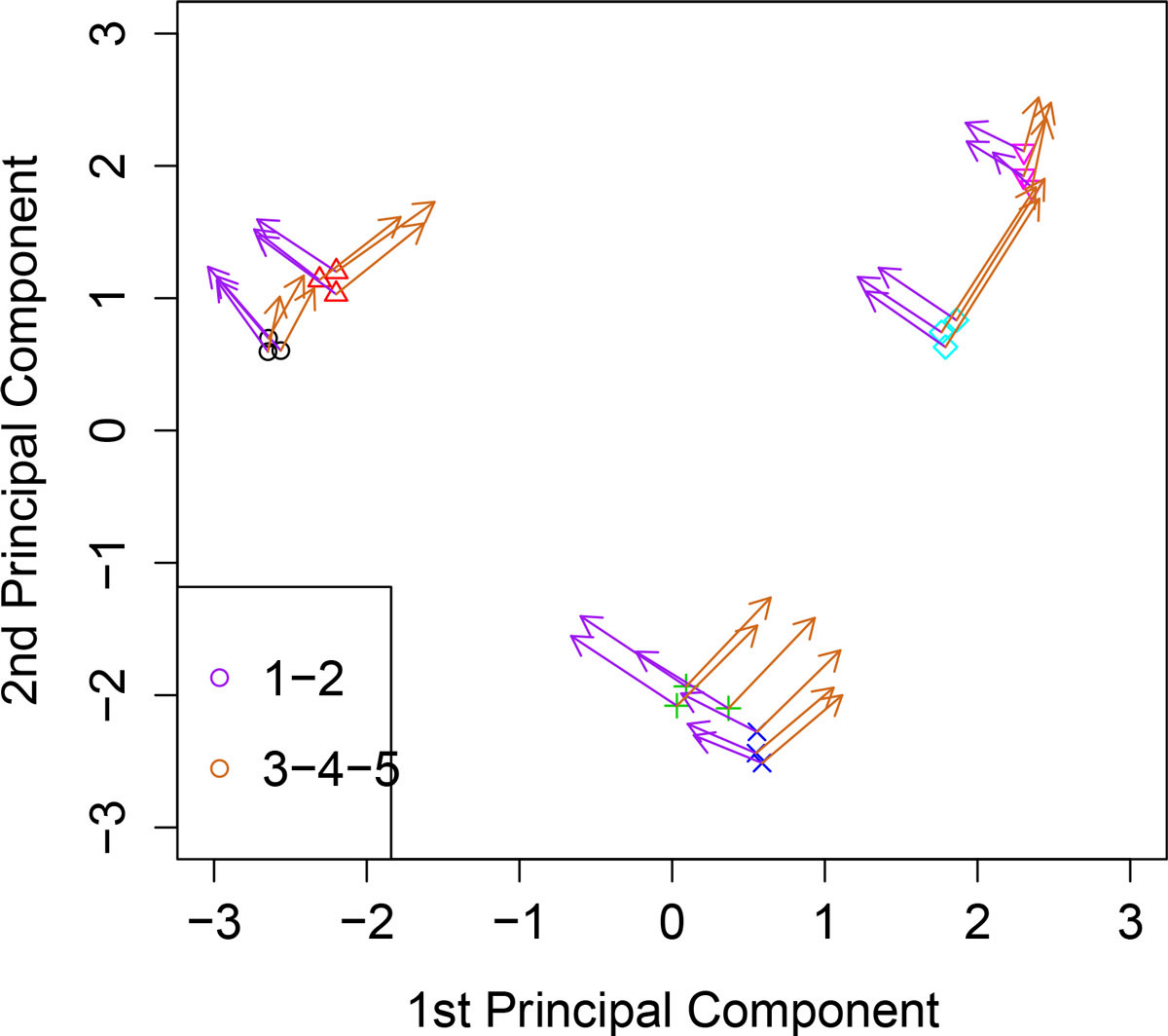
**Kernel PCA analyzing the toy example**. Linear combinations of variables are represented by vectors that indicate the direction of maximum growth in each of the linear combinations.

A natural extension of the above procedure is the representation of linear combinations of input variables. Details can be found in section 3.2. With the aim to show this property we displayed in Figure 3 the samples and the linear combinations *X*_1 _+ *X*_2 _and *X*_3 _+ *X*_4 _+ *X*_5 _at each sample. Linear combinations are represented by vectors that point to the direction of maximum growth in each of the linear combinations. We can observe that at each sample vectors point to those groups with higher values in each of linear combinations. For example, vectors representing *X*_1 _+ *X*_2 _point to group 1, and vectors representing *X*_3 _+ *X*_4 _+ *X*_5 _point to group 3.

#### Analyzing the nutrigenomic dataset

**Figure 4:**
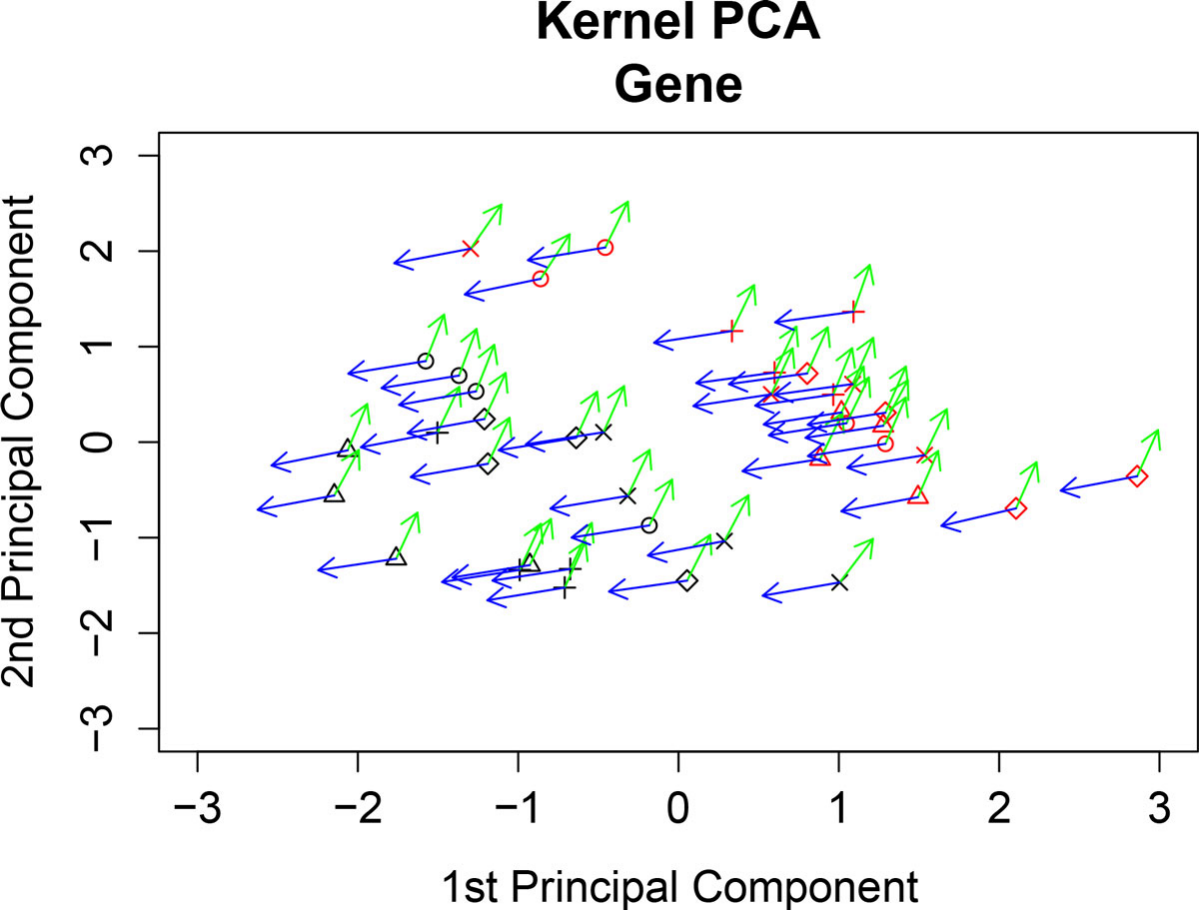
**Kernel PCA of gene expression**. The genes *AOX *(blue vector) and *CAR1 *(green vector) are represented at each sample point. WT samples are represented in black and PPAR samples in red. Diet representation is: (ref) diet by the letter x; (coc) diet by circles; (sun) diet by diamonds; (lin) diet by plus signs; and (fish) diet by triangles.

**Figure 5:**
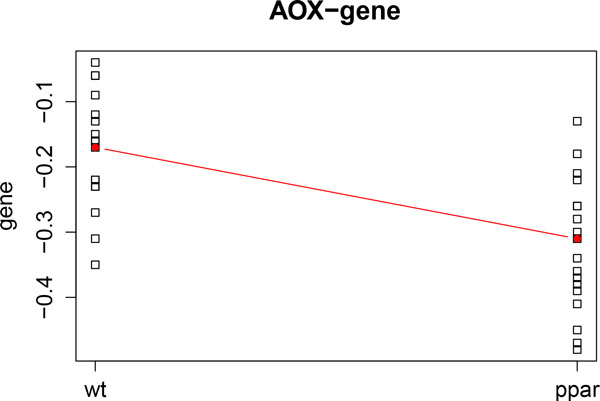
**AOX gene profile**. Profile of the median gene expression of the *AOX *gene.

**Figure 6:**
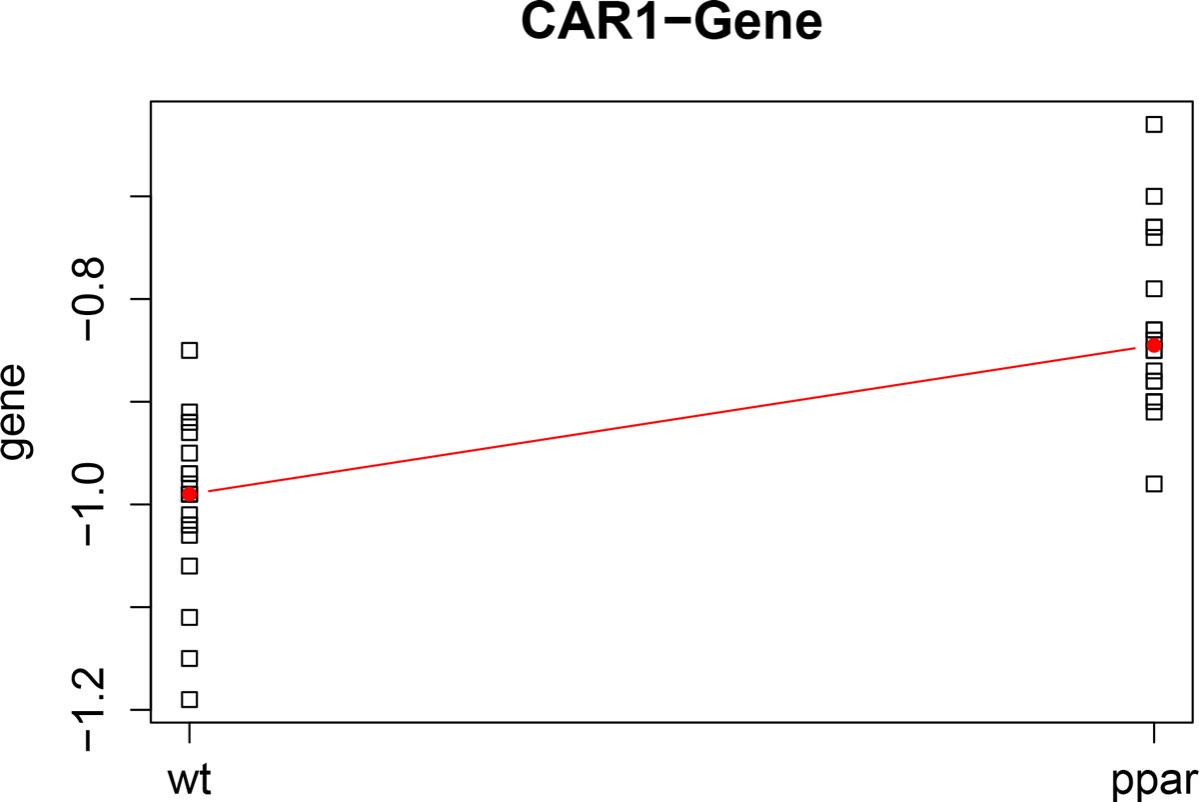
**CAR1 gene profile**. Profile of the median gene expression of the *CAR1 *gene.

We illustrate the representation of variables by analyzing the dataset in [11]. We apply kernel PCA and representation of variables to the genomic data and FA data. Firstly, we compute kernel PCA by analyzing only gene expression level data. Figure 4 shows the two leading axes of kernel PCA. We can observe that the genotypes are clearly separated (WT samples are represented in black and PPAR samples in red). Diet representation is: ref diet is represented by the letter x; coc diet by circles; sun diet by diamonds; lin diet by plus signs; and fish diet by triangles). Figure 4 shows the *AOX *(blue vector) and *CAR1 *(green vector) genes. Vectors indicate the direction of maximum growth of the gene expression at each sample point. Thus, we can observe that *AOX *increases towards WT and *CAR1 *towards PPAR. These results are in agreement with those found in [11] and [12]. Figure 5 and Figure 6 show the profiles of the medians of the expression of *AOX *and *CAR1 *grouped by genotype. We can observe that these profiles agree with the kernel PCA representation.

**Figure 7:**
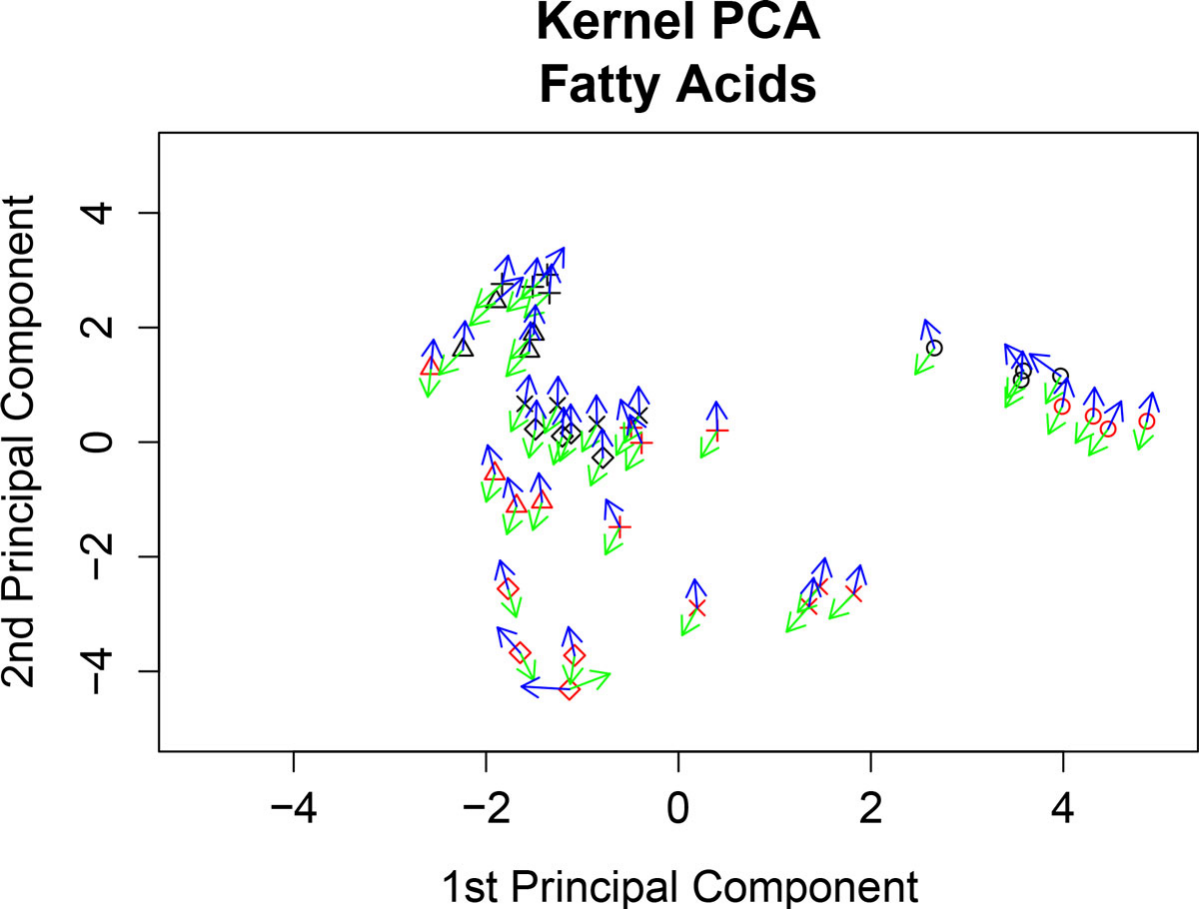
**Kernel PCA of fatty acid concentrations**. The fatty acids C16.0 (blue vector) and C20.2*ω*.6 (green vector) are represented at each sample point. WT samples are represented in black and PPAR samples in red. Diet representation is: (ref) diet by the letter x; (coc) diet by circles; (sun) diet by diamonds; (lin) diet by plus signs; and (fish) diet by triangles.

Secondly, to compare results, we compute kernel PCA analyzing only FA levels. In Figure 7 we can observe that the sample points are separated by genotype, but we can also observe that the samples with coc diet (a diet with hydrogenated coconut oil as a saturated FA diet) form a cluster. Figure 7 shows C20.2*ω*.6 (green vector) and C16.0 (blue vector) FAs. It reveals higher levels of C20.2*ω*.6 towards PPARα-deficient clustered samples (red) and that levels of C16.0 are higher towards the WT cluster of samples (black).

**Figure 8:**
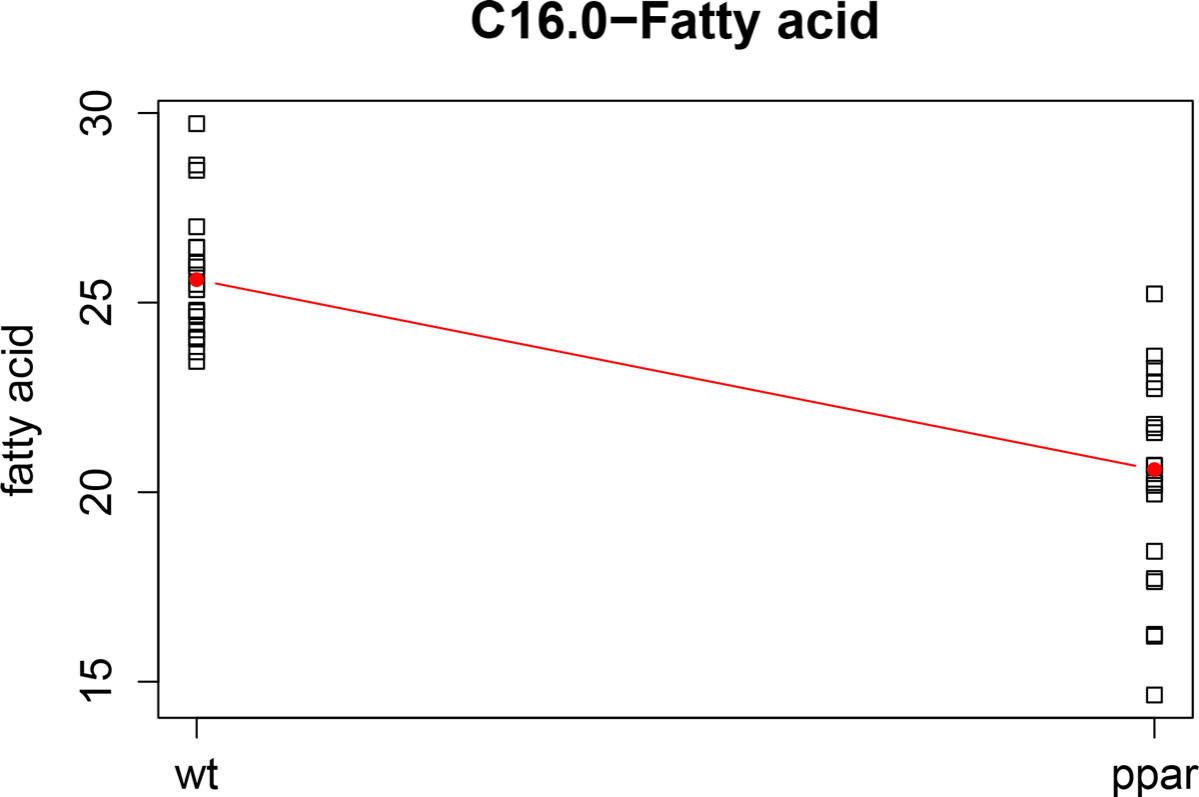
**C16.0 fatty acid profile**. Profile of the median concentrations of the C16.0 fatty acid.

**Figure 9:**
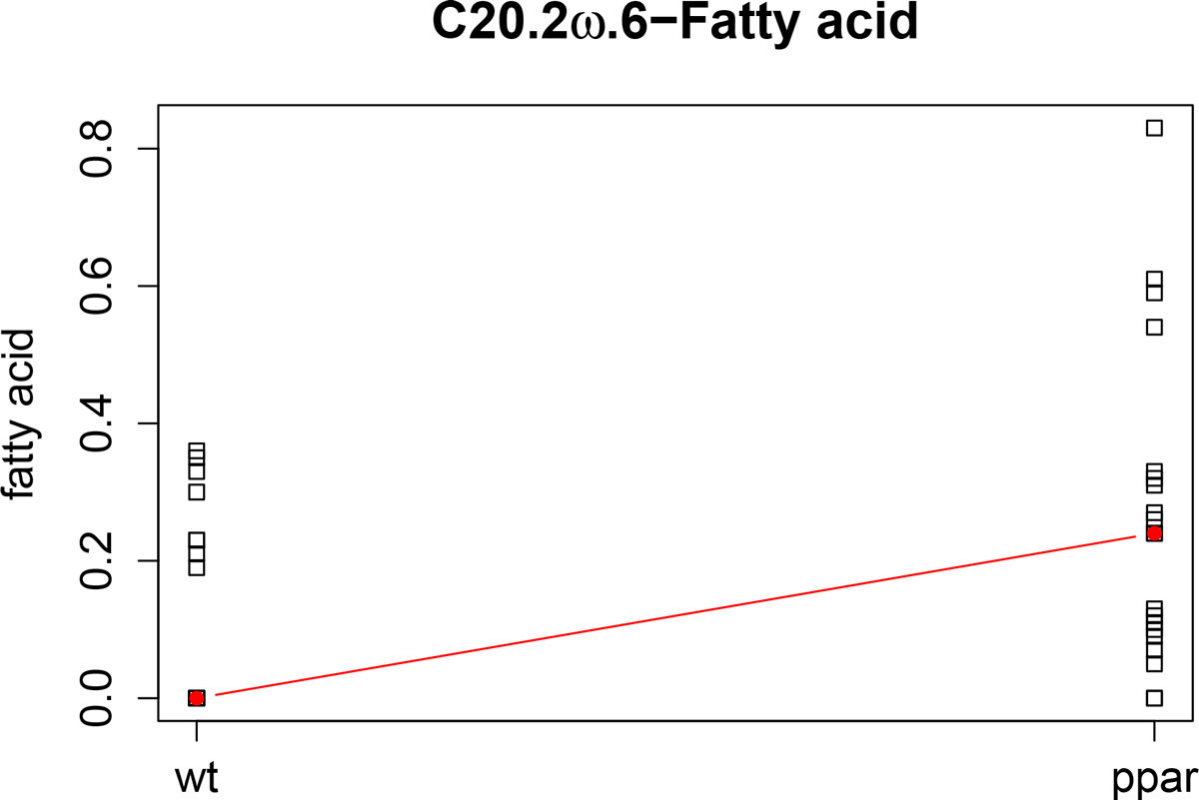
**C20.2*ω*.6 fatty acid profile**. Profile of the median concentrations of the C20.2*ω*.6 fatty acid.

These results are also in agreement with those found in [11] and [12]. Figure 8 and Figure 9 show the profiles of the medians of the concentrations of C16.0 and C20.2*ω *FAs, grouped by genotype. We can observe that these profiles agree with the kernel PCA representation.

### Data integration and representation of input variables

The kernel formalism allows us to combine heterogeneous datasets for data fusion. Basic algebraic operations such as addition, multiplication and exponentiation preserve the key properties of symmetry and positive semidefiniteness, and thus allow a simple but powerful algebra of kernels. If *k*_1 _and *k*_2 _are kernels defined respectively on $X_{1}\;\times X_{1}\;$ and $X_{2}\;\times X_{2}\;$, then their direct sum:

$$\left(k_{1}⊕k_{2}\right)\left(x_{1},x_{2},x_{1}^{\prime },x_{2}^{\prime }\right)=k_{1}\left(x_{1},x_{1}^{\prime }\right)+k_{2}\left(x_{2},x_{2}^{\prime }\right)$$

is a kernel on $\left(X_{1}\;\times X_{2}\right)\;\times \;\left(X_{1}\;\times X_{2}\right).$ Here, $x_{1},x_{1}^{\prime }\in X_{1}$ and $x_{2},x_{2}^{\prime }\in X_{2}$.

This construction can be useful if the different parts of the input have different meanings and should therefore be dealt with differently. In that case, we can split the inputs into two parts, **X**_1 _and **X**_2_, and use two different kernels for these parts. This is the case when we are integrating two separate datasets. In consequence, our procedure can easily be extended to data fusion. Firstly, we reduce the dimension of the entire data (**x**_1*i*_, **x**_2*i*_), *i *= 1, ..., *m*, by applying kernel PCA with the kernel *K *given by *k*_1 _⊕ *k*_2_. Secondly, to find the coordinates of a test point:

$$y=\left(y_{1},y_{2}\right),$$

we proceed by analogy with (8), so that (7) becomes:

$$Z={\left(K\left(y_{1},x_{1i},y_{2},x_{2i}\right)\right)}_{m\;\times \;1}={\left(k_{1}\left(y_{1},x_{1i}\right)+k_{2}\left(y_{2},x_{2i}\right)\right)}_{m\;\times \;1}.$$

When we integrate two datasets, we can represent any given input variable that belongs to one of the datasets. Let us suppose that we wish to represent the variable $X_{k}^{l}$ that belongs to the dataset *l *= 1, 2. Then (2) becomes:

$${\left(\frac{dZ_{s}^{i}}{ds}\right|}_{s=0}\;=\;{\left(\frac{\partial K_{l}\left(y_{l},x_{li}\right)}{\partial y_{lk}}\right|}_{y_{l}=a_{l}}.$$

Then, formula (1) allows us to display variables that belong to any of the datasets over the kernel PCA representation of samples, simultaneously.

#### Analyzing the nutrigenomic dataset

**Figure 10:**
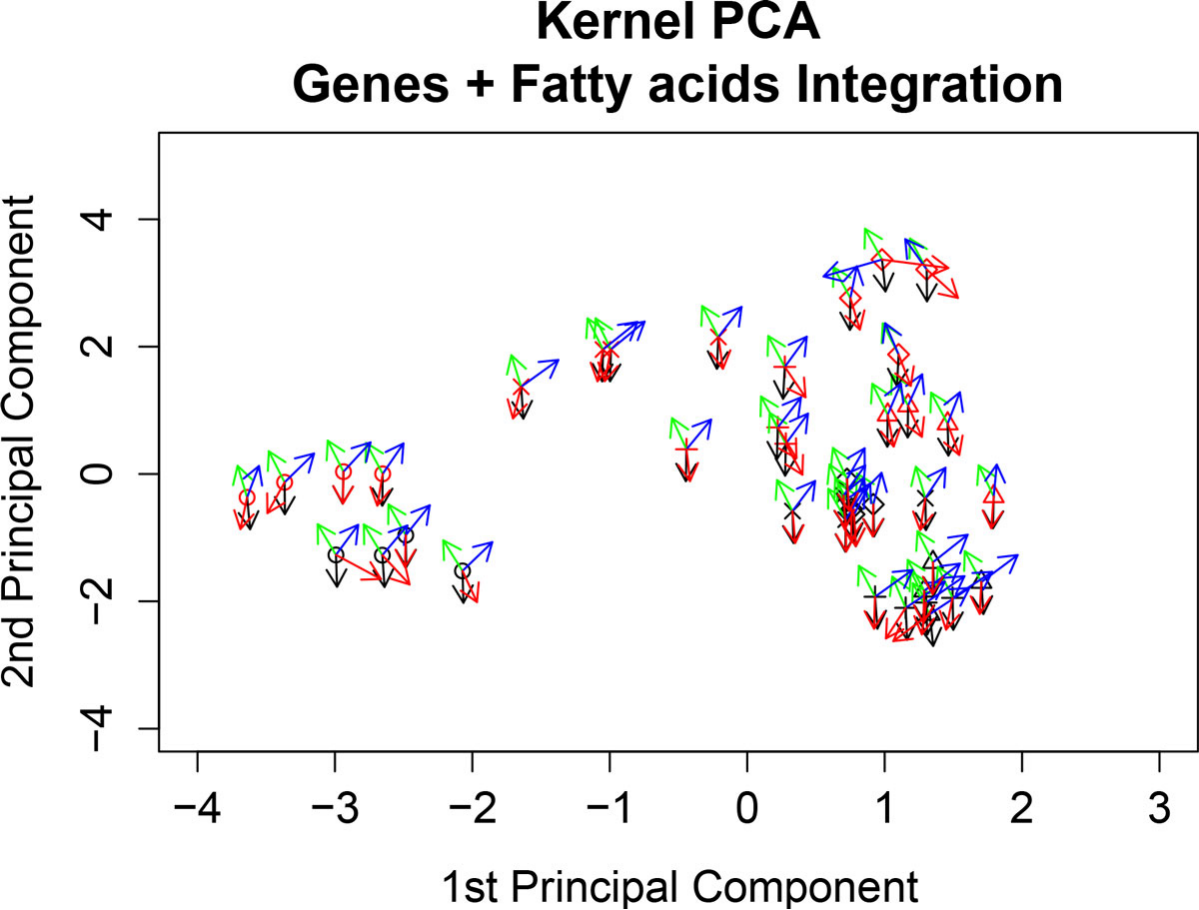
**Kernel PCA analyzing gene expression and fatty acid concentrations simultaneously**. The genes AOX (black vector) and CAR1 (green vector) and fatty acids C20.2*ω*.6 (blue vector) and C16.0 (red vector) are represented at each sample point. The WT samples are represented in black and the PPAR samples in red. Diet representation is: (ref) diet by the letter x; (coc) diet by circles; (sun) diet by diamonds; (lin) diet by plus signs; and (fish) diet by triangles.

Continuing with the same nutrigenomic study, we compute kernel PCA by analyzing both datasets simultaneously; that is, gene expressions and FA concentrations. We observe that the genotypes are clearly separated (WT is represented in black and PPAR in red) and also mice with the coc diet form a cluster of both genotypes; see Figure 10. Also, Figure 10 shows AOX (black vector) and CAR1 (green vector) genes, and C20.2*ω*.6 (blue vector) and C16.0 (red vector) FAs. It reveals higher expression of CAR1 and higher concentrations of C20.2*ω*.6 towards the PPAR cluster. In contrast, AOX gene expression and concentrations of C16.0 are higher towards the WT cluster. These results are in agreement with those found in the individual kernel PCAs above.

### Representation of linear combinations of input variables

A natural extension of the above procedure is the representation of linear combinations of input variables. This may be useful for representing gene modules or gene networks. Let us suppose that we wish to represent the linear combination: $X_{k_{1}}+X_{k_{2}}+\cdots +X_{k_{l}}$, where *k*_1_*, k*_2_*,...,k_l _*∈{1, 2*, ..., n*}, with *ki ≠ kj , i,j *= 1*, ..., l*. Then, when *K *is the Gaussian radial basis function kernel, (2) becomes:

$${\left(\frac{dZ_{s}^{i}}{ds}\right|}_{s=0}\;={\left(\overset{l}{\underset{t=1}{\sum }}\frac{\partial K\left(y,x_{i}\right)}{\partial y_{k_{t}}}\right|}_{y=a}.\;$$

Then, formula (1) allows us to represent any linear combination of input variables.

#### Analyzing the nutrigenomic dataset

**Figure 11:**
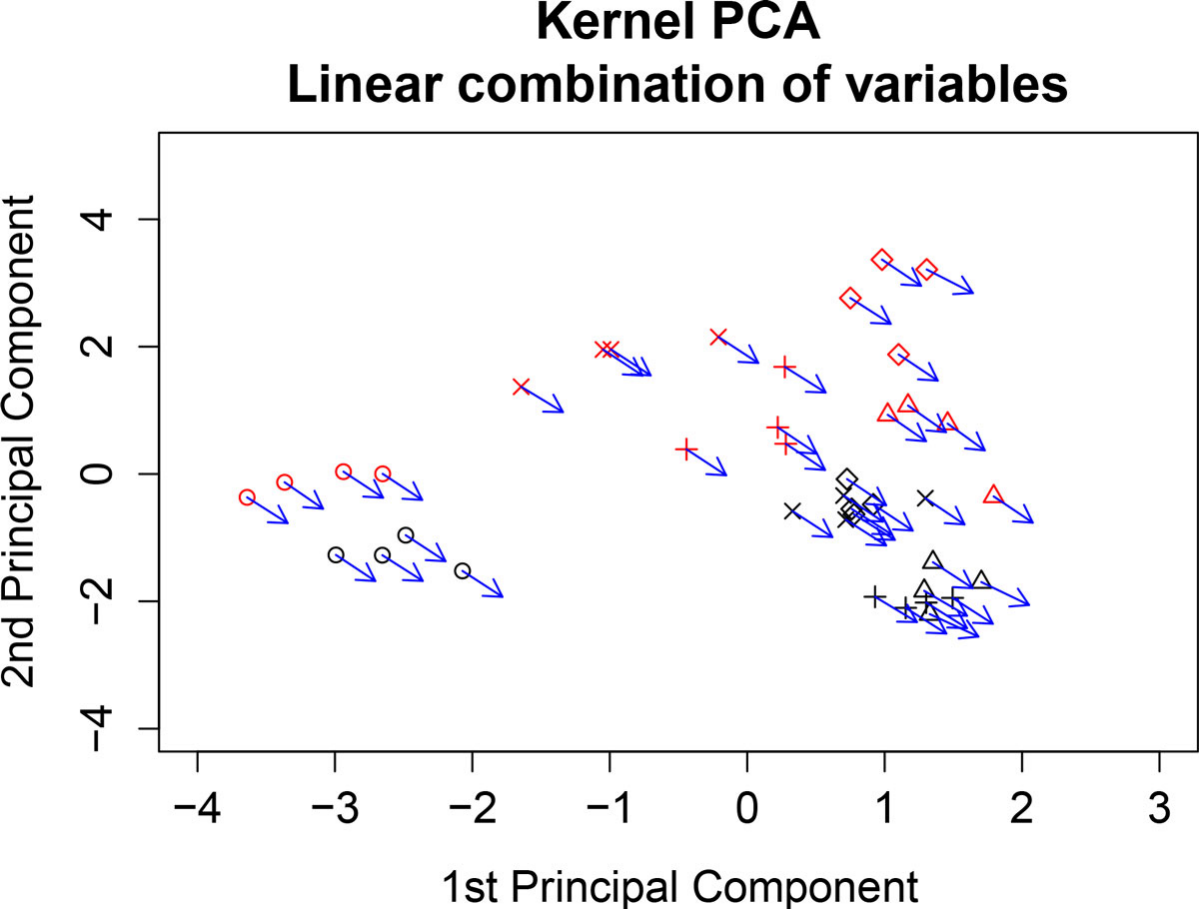
**Representation of linear combinations of input variables**. The sum of the expression of the genes: GSTpi2, CYP3A11 and CYP2c29 is represented. These genes are associated with detoxification. Wild type samples are represented in black and PPAR samples in red. Diet representation is: (ref) diet by the letter x; (coc) diet by circles; (sun) diet by diamonds; (lin) diet by plus signs; and (fish) diet by triangles.

To illustrate this procedure we have analyzed the genes GSTpi2, CYP3A11 and CYP2c29. These genes are involved in the functioning of detoxification [12]. We perform kernel PCA analyzing both dataset simultaneous and represent the sum of the expressions of the genes GSTpi2, CYP3A11 and CYP2c29. Figure 11 shows sample points and the vector corresponding to the sum of the three gene expressions is attached to each point. The vector indicates the direction of maximum growth of the sum of the expressions. We observe that the sum of the expressions increases towards the fish diet. This is in agreement with the findings in [12].

### Revealing the interpretability of input variables

Our procedure for representing input variables on the two-dimensional subspace expanded by the two main eigenvectors of $\tilde{C}$, displays the variables as vectors whose direction is the direction of maximum growth of the variable at a given point; in particular, at the sample points.

So, if we set a direction in this plane, given by a vector *w*, we can search for input variables whose representation on the kernel PCA plane are correlated with this direction. Let us suppose that we observe clusters of samples in the kernel PCA representation; then an interesting direction can be given by the vector defined by any two cluster centroids.

Once we have selected a vector *w*, we denote *w_i _a*s the parallel vector of *w *attached to the image given by kernel PCA of the sample point **x***_i_*, *i = *1*, ..., m*. For any variable *X_k_*, we now compute its vector representation in kernel PCA using formula (1); we denote this vector as ${\left(\frac{d\sigma^{k}}{ds}\right|}_{s=0}$. Therefore, for each sample point, **x***_i_*, *i *= 1, ..., *m*, we have two vectors, one corresponding to the direction *w_i_*, and other corresponding to the *X_k _*representation, ${\left({\left(\frac{d\sigma^{k}}{ds}\right|}_{s=0}\right)}_{x_{i}}$. After this, to measure the strength of the correlation between *X_k _*and *w*, we average the cosine of the angles between each pair of vectors, that is:

$$R_{k}:=\frac{1}{m}\overset{m}{\underset{i=1}{\sum }}\cos\left(w_{i},{\left({\left(\frac{d\sigma^{k}}{ds}\right|}_{s=0}\right)}_{x_{i}}\right).$$

Finally, we order all the variables according to *R_k _*and we can select those with higher values and also those with lower values. Thus, in this way, for each sample cluster, we can find the correlated variables with higher and lower values. Knowledge of such variables can improve the biological interpretability of the results.

A natural extension of this procedure is to take as *w *the vector corresponding to one of the input variables. Then, if we know that a certain input variable is useful for interpreting the kernel PCA representation, we can search for other input variables whose representation on the kernel PCA plane are correlated with this feature. If we are integrating multiple datasets, we can search for correlated variables in each dataset.

#### Analyzing the nutrigenomic dataset

**Figure 12:**
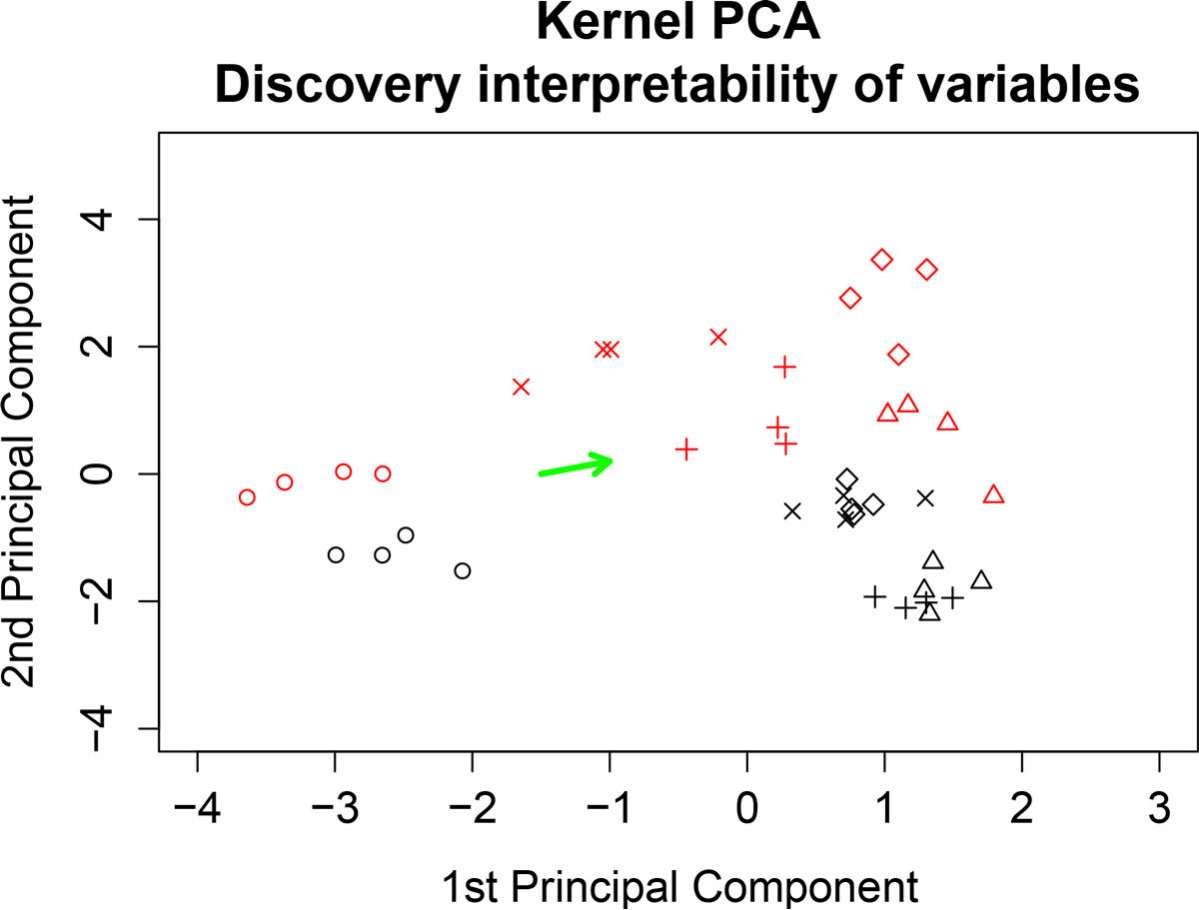
**Kernel PCA analyzing gene expression and fatty acid concentrations simultaneously**. The green vector represents variables that are expressed less in samples with the coc diet. It is defined by two cluster centroids: the left-hand cluster is the coc diet; and the right-hand cluster is comprised of the other diets.

To illustrate this procedure. We have selected a preferred direction in the kernel PCA plane. Figure 12 shows this direction (green vector). This direction represents variables that are less expressed in samples with the coc diet than in those with other diets. Tables 1 and 2 summarize the genes and FAs that are most correlated with the selected direction.

**Table 1:** Fatty acids: correlation with the preferred direction.

FA	mean	sd
C16.1*ω*.7	-0.927	0.100
C20.3*ω*.9	-0.917	0.336
C18.1*ω*.7	-0.907	0.270
C14.0	-0.898	0.131
C18.3*ω*.6	-0.862	0.372
C18.1*ω*.9	-0.695	0.132
C16.1*ω*.9	-0.480	0.224
C16.0	-0.295	0.265
C20.1*ω*.9	0.176	0.401
C22.5*ω*.3	0.198	0.346
C20.3*ω*.3	0.235	0.383
C20.5*ω*.3	0.300	0.219
C20.3*ω*.6	0.386	0.227
C18.0	0.392	0.171
C22.6*ω*.3	0.453	0.151
C20.2*ω*.6	0.601	0.306
C20.4*ω*.6	0.664	0.360
C22.4*ω*.6	0.684	0.367
C18.2*ω*.6	0.718	0.290
C18.3*ω*.3	0.727	0.482
C22.5*ω*.6	0.731	0.499

Fatty acids. Mean and standard deviation of the *Rk *measure of the strength of correlation with the preferred direction.

**Table 2:** Genes: correlation with the preferred direction.

gene	mean	sd
S14	-0.998	0.002
ACC2	-0.997	0.004
LPL	-0.997	0.005
ap2	-0.996	0.006
NGFiB	-0.996	0.005
i.FABP	-0.995	0.007
COX1	-0.993	0.012
CIDEA	-0.993	0.012
MDR1	-0.991	0.016
Lpin	-0.991	0.007
MTHFR	-0.991	0.012
Lpin1	-0.989	0.009
i.BAT	-0.988	0.014
PPARg	-0.986	0.025
ACAT2	-0.984	0.013
CYP2b10	-0.978	0.022
hABC1	-0.976	0.021
ACC1	-0.975	0.012
SPI1.1	0.353	0.042
GSTpi2	0.587	0.038

Gene codes. Mean and standard deviation of the *R_k _*measure of the strength of correlation with the preferred direction.

In Table 1, we can observe that FAs with negative correlation, such as C16.1*ω*.7, C20.3*ω*.9 and C18.1*ω*.7, represent FAs with higher concentrations in samples with the coc diet. In contrast, FAs that are positively correlated, such as C22.4*ω*.6, C18.2*ω*.6, C18.3*ω*.3 and C22.5*ω*.6, represent FAs with higher concentrations in samples with other types of diet. Furthermore, in Table 2, we can observe that genes with negative correlation at the top of the table, such as S14, ACC2 and LPL, are more highly expressed in samples with the coc diet, whereas genes at the bottom of the table, that are positively correlated, are less expressed in the coc diet samples. These results are in agreement with those found in [12].

## Conclusions

With the rapidly increasing amount of genomic, proteomic, and other high-throughput data that is available, the importance of data integration has increased significantly recently. Biologists, medical scientists, and clinicians are also interested in integrating the high-throughput data that has recently become available with previously existing clinical, laboratory and biological information.

Kernel methods, in particular kernel PCA, constitute a powerfully methodology because they allow us to reduce dimensionality and integrate multiple datasets, simultaneously. Moreover, in this paper we have introduced a set of procedures to improve the interpretability of kernel PCA representations. The procedures are related to the following aspects: 1) representation of variables; 2) linear combination of representations of variables; 3) data integration and representation of variables; and 4) revealing the interpretability of input variables. Our procedure is a kernel-based exploratory tool for data mining that enables us to extract nonlinear features while representing variables.

## Methods

Given a sample space  X, a real valued positive definite kernel *k *on  X is a map k:X×X→ℝ such that $k\left(x,y\right)=k\left(y,x\right),\sum_{i,j=1}^{m}\alpha_{i}\alpha_{j}k\left(x_{i},x_{j}\right)\geq 0$ for all $m\in \mathbb{N},\alpha_{i}\in \mathbb{R},x_{i}\in X\;i=1,\ldots ,m$, and kernel is zero is attained if all the coefficients α*_j _*are zero. A kernel can be interpreted as a similarity measure of the samples and allow us to identify each x∈Xwith a real function given by

$$\begin{array}{ccc} \phi :X & \to {\mathbb{R}}^{X}=\left\{f:X\to \mathbb{R}\right\} & \\ x & \mapsto \phi \left(x\right)\left(\cdot \right)=k\left(\cdot ,x\right) \end{array}$$

which is an element of a dot product vector space that will be called feature space [5]. It consists of all functions

$$f\left(\cdot \right)=\overset{m}{\underset{i=1}{\sum }}\alpha_{i}k\left(\cdot ,\;x_{i}\right)$$

for any $m\in \mathbb{N}\;\text{and}\;x_{1},\ldots ,x_{m}\in X,\alpha_{1},\ldots ,\alpha_{m}\in \mathbb{R}$. It has the reproducing property

<k(⋅,x),f>=f(x)

Implying $\left\langle \phi \left(x\right),\phi \left(y\right)\right\rangle =\left\langle k\left(\cdot ,x\right),k\left(\cdot ,y\right)\right\rangle =k\left(x,y\right)$. After completion we can turn our feature space into a Hilbert space ℋ*_k _*[5]. The space ℋ*_k _*is the *reproducing kernel Hilbert space *(RKHS) induced by the kernel function *k*.

Given any *ϕ *and any set of observations ***x***_1_*, ..., **x**_m_*, the Gram or kernel matrix of *k *with respect ***x***_1_, ..., ***x**_m _*is the *m × m *matrix *K *with elements $K_{ij}=\left\langle \phi \left(x_{i}\right),\phi \left(x_{j}\right)\right\rangle =k\left(x_{i},x_{j}\right)$. Let us define

$$\bar{\phi }:=\frac{1}{m}\overset{m}{\underset{i=1}{\sum }}\phi \left(x_{i}\right)$$

then, the points

(3) $$\tilde{\phi }\left(x_{i}\right)=\phi \left(x_{i}\right)-\bar{\phi }$$

will be centered. Let $\tilde{K}$ be denote the kernel matrix of centered points, ${\tilde{K}}_{ij}=\left\langle \tilde{\phi }\left(x_{i}\right),\tilde{\phi }\left(x_{j}\right)\right\rangle$, Because we do not have the centered data (3), we cannot compute $\tilde{K}$ explicitly, however we can express it in terms of its noncentered counterpart *K *[5]. Using the vector **1***_m _*= (1, ..., 1)^T^, we get the more compact expression

$$\tilde{K}=K-\frac{1}{m}K1_{m}1_{m}^{T}-\frac{1}{m}1_{m}1_{m}^{T}K+\frac{1}{m^{2}}\left(1_{m}^{T}K1_{m}\right)1_{m}1_{m}^{T}.$$

In ℋ*_k _*the covariance matrix takes the form

$$\tilde{C}=\frac{1}{m}\overset{m}{\underset{j=1}{\sum }}\tilde{\phi }\left(x_{j}\right)\;\tilde{\phi }{\left(x_{j}\right)}^{T}.$$

We have to find eigenvalues $\tilde{\lambda }\geq 0$ and nonzero eigenvectors $\tilde{V}\in H_{k}\backslash \left\{0\right\}$ satisfying

(4) $$\tilde{C}\tilde{V}=\tilde{\lambda }\tilde{V}$$

To find the solutions of (4) we solve the dual eigenvalue problem

(5) $$\tilde{K}\tilde{\alpha }=m\tilde{\lambda }\tilde{\alpha },$$

with $\tilde{\alpha }$ being the expansion coefficients of an eigenvector (in ℋ*_k_*) in terms of the centered points (3)

(6) $$\tilde{V}=\overset{m}{\underset{i=1}{\sum }}{\tilde{\alpha }}_{i}\tilde{\phi }\left(x_{i}\right).$$

The solution ${\tilde{\alpha }}^{k},k=1,...,r,$ are normalized by normalizing the corresponding vector ${\tilde{V}}^{k}$ in ℋ*_k_*, which translates into ${\tilde{\lambda }}_{k}\left\langle {\tilde{\alpha }}^{k},{\tilde{\alpha }}^{k}\right\rangle =1$.

Consider a test point ***y***. To find its coordinates we compute projections of centered *ϕ*-images of ***y ***onto the eigenvectors of the covariance matrix of the centered points,

$$\begin{array}{ccc} \left\langle \tilde{\phi }\left(y\right),{\tilde{V}}^{k}\right\rangle & =\left\langle \phi \left(y\right)-\tilde{\phi },{\tilde{V}}^{k}\right\rangle & \\ & =\overset{m}{\underset{i=1}{\sum }}{\tilde{\alpha }}_{i}^{k}\left\langle \phi \left(y\right)-\bar{\phi },\phi \left(x_{i}\right)-\bar{\phi }\right\rangle \\ & =\overset{m}{\underset{i=1}{\sum }}{\tilde{\alpha }}_{i}^{k}\left(\left\langle \phi \left(y\right),\phi \left(x_{i}\right)\right\rangle -\left\langle \bar{\phi },\phi \left(x_{i}\right)\right\rangle -\left\langle \phi \left(y\right),\tilde{\phi }\right\rangle +\left\langle \bar{\phi },\bar{\phi }\right\rangle \right)\\ & =\overset{m}{\underset{i=1}{\sum }}{\tilde{\alpha }}_{i}^{k}\left\{K\left(y,x_{i}\right),-,\frac{1}{m},\overset{m}{\underset{s=1}{\sum }},K,\left(x_{s},x_{i}\right)\right)\\ & \left(\;\;\;\;-\frac{1}{m}\overset{m}{\underset{s=1}{\sum }}K\left(y,x_{s}\right)+\frac{1}{m^{2}}\overset{m}{\underset{s=1}{\sum }}K\left(x_{s},x_{t}\right)\right\}. \end{array}$$

Introducing the vector

(7) $$Z={\left(K\left(y,x_{i}\right)\right)}_{m\times 1}.$$

Then,

(8) $$\begin{array}{ccc} {\left(\left\langle \tilde{\phi }\left(y\right),{\tilde{V}}^{k}\right\rangle \right)}_{1\times r} & =Z^{T}\tilde{V}-\frac{1}{m}1_{m}^{T}K\tilde{V}-\frac{1}{m}\left(Z^{T}1_{m}\right)1_{m}^{T}\tilde{V}+\frac{1}{m^{2}}\left(1_{m}^{T}K1_{m}\right)1_{m}^{T}\tilde{V} & \\ & =Z^{T}\left(I_{m}-\frac{1}{m}1_{m}1_{m}^{T}\right)\tilde{V}-\frac{1}{m}1_{m}^{T}K\left(I_{m}-\frac{1}{m}1_{m}1_{m}^{T}\right)\tilde{V}\\ & =\left(Z^{T}-\frac{1}{m}1_{m}^{T}K\right)\left(I_{m}-\frac{1}{m}1_{m}1_{m}^{T}\right)\tilde{V}, \end{array}$$

where $\tilde{V}$ is a *m × r *matrix whose columns are the eigenvectors ${\tilde{V}}^{1},\ldots ,{\tilde{V}}^{r}$.

## Competing interests

The authors declare that they have no competing interests.

## Authors' contributions

FR suggest the combination of Kernel PCA and variable representation and designed the study. JMO was responsible for the development of the procedure. EV implemented the approach, coded the procedures and prepared analysis and results. FR and EV wrote the manuscript.

## Declarations

The publication costs for this article were partially funded by MEC-MTM2008-00642.
